# A case of synchronous advanced gastric cancer and locally advanced prostate cancer with combined laparoscopic and robotic surgery: A case report

**DOI:** 10.1016/j.ijscr.2019.02.032

**Published:** 2019-03-01

**Authors:** Toru Imagami, Satoru Takayama, Taku Hattori, Ryohei Matsui, Masaki Sakamoto, Hisanori Kani, Satoshi Kurokawa, Tsuyoshi Fujiwara

**Affiliations:** a Department of Surgery, Nagoya Tokushukai General Hospital, Kasugai City, Japan; b Department of Urology, Nagoya Tokushukai General Hospital, Kasugai City, Japan

**Keywords:** GC, gastric cancer, PCa, prostate cancer, LTG, laparoscopic total gastrectomy, RARP, robotic-assisted radical prostatectomy, ADT, androgen deprivation therapy, PSA, prostate-specific antigen, CT, computed tomography, MRI, magnetic resonance imaging, Synchronous cancer, Gastric cancer, Combined laparoscopic surgery, Multidisciplinary treatment for synchronous cancer, Case report

## Abstract

**Introduction:**

The optimal management strategy for synchronous gastric cancer (GC) and prostate cancer (PCa) remains unclear, particularly in cases in which two cancers are progressive.

**Presentation of case:**

A 68-year-old man diagnosed with synchronous advanced GC and locally advanced PCa was referred to our institution. Laparoscopic total gastrectomy (LTG) and robotic-assisted radical prostatectomy were simultaneously performed. The postoperative course was similar to the standard postoperative course of LTG alone. Pathological diagnoses were T3N3aM0 gastric adenocarcinoma and T3N0M0 prostatic adenocarcinoma. Adjuvant chemotherapy and adjuvant androgen deprivation therapy (ADT) for GC and PCa were initiated on postoperative days 15 and 27, respectively. Six months subsequent to surgery, the patient received adjuvant chemotherapy and ADT, and no evidence of cancer recurrence was observed.

**Discussion:**

In terms of survival, curative resection with adjuvant therapy is advantageous for patients with advanced GC or locally advanced PCa. At present, treatment for synchronous cancer should be combined with optimal management for individual cancers. Minimally invasive surgery may play an important role in the multidisciplinary treatment of synchronous advanced cancer.

**Conclusion:**

Combined laparoscopic and robotic surgery for synchronous GC and PCa allows for minimally invasive radical resection and appropriate adjuvant therapy.

## Highlights

• The optimal management for synchronous advanced cancer remains controversial.
• Multidisciplinary treatment strategies are important for synchronous advanced cancer.
• Combined laparoscopic and robotic surgery allows minimally invasive resection.
• Simultaneous endoscopic surgery is recommended for synchronous advanced cancer.

## 1 Introduction

In Japan, the incidence rate of synchronous gastric cancer (GC) and prostate cancer (PCa) is 2% [1,2]. Most synchronous cancers are detected during preoperative workup [3]. Improvement in preoperative diagnostic techniques may increase the diagnostic frequency of synchronous GC and PCa. However, an optimal management strategy for synchronous GC and PCa remains unclear, particularly in cases in which two cancers are progressive.

Recently, endoscopic surgery has been widely used for GC or PCa. In contrast, only few studies on simultaneous endoscopic surgery for synchronous GC and PCa have been reported [1]. At our center, laparoscopic total gastrectomy (LTG) combined with robotic-assisted radical prostatectomy (RARP) is performed for synchronous GC and PCa. Postoperative therapy is applied for each cancer. In this study, a multidisciplinary treatment strategy for synchronous advanced GC and locally advanced PCa is presented. In addition, a pertinent literature review was conducted. This work has been reported in line with the SCARE criteria [4].

## 2 Presentation of case

**Fig. 1:**
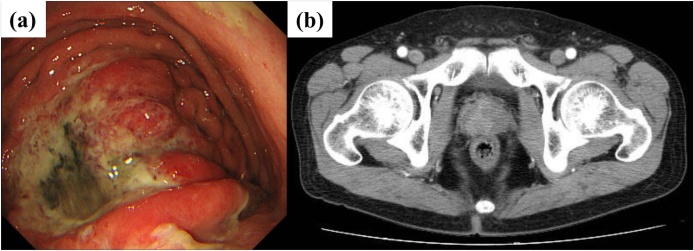
Preoperative image findings. (a) Gastroscopy revealed a type 3 tumor on the greater curvature of the stomach. (b) Prostate cancer was suspected to spread out of the capsule by contrast-enhanced CT.

**Fig. 2:**
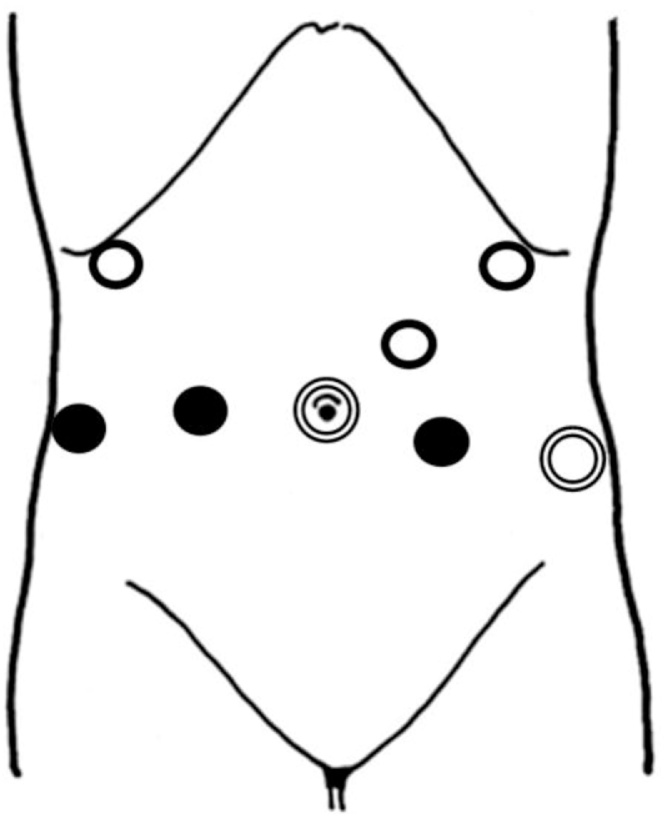
Port arrangement (: 12 mm trocar, ●: 8 mm trocar, ○: 5 mm trocar). Surgeons inserted additional two 5 mm trocars on left and right upper abdomen.

A 68-year-old male with an unremarkable medical history was referred to our hospital for the screening of GC and PCa. His physical examination result was not significant. Gastroscopy revealed a type 3 tumor on the greater curvature of the stomach (Fig. 1a). He was pathologically diagnosed with gastric adenocarcinoma (tub2). Contrast-enhanced computed tomography (CT) scan revealed metastasis to the peri-gastric lymph nodes. However, no evidence of distant metastasis was observed. According to the screening results for PCa, the serum concentration of prostate-specific antigen (PSA) increased to 26 ng/mL. Magnetic resonance imaging (MRI) and prostatic biopsy revealed prostatic adenocarcinoma, with a Gleason score of 4–5. Based on CT scan and MRI findings, local progression of PCa was observed (Fig. 1b). The adenocarcinomas were pathologically diagnosed as separate cancers. His preoperative diagnosis was synchronous T3N2M0 gastric adenocarcinoma and T3N0M0 prostatic adenocarcinoma. According to the Japanese guideline, neoadjuvant chemotherapy (NAC) was not recommended. His prognosis based on GC, and GC treatment, not PCa treatment, should be prioritized. Based on consultation from surgeons and urologists, we considered the following options: (1) simultaneous surgery followed by adjuvant chemotherapy and (2) surgery and adjuvant chemotherapy for GC followed by prostatectomy. According to our previous experience, simultaneous resection of GC and PCa was proposed. The patient agreed with our decision and underwent LTG combined with RARP. The port arrangement is depicted in Fig. 2.

**Fig. 3:**
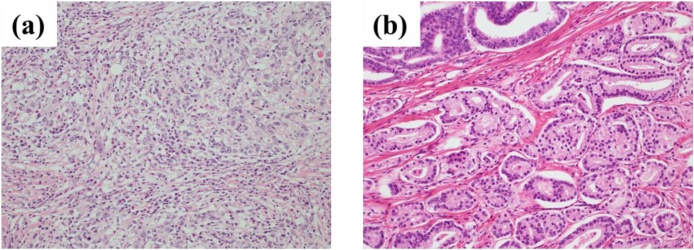
Pathological findings are shown. Each cancer was pathologically diagnosed as separate adenocarcinoma. (a) Gastric cancer; Adenocarcinoma, U, Gre, type 2, 40 mm × 40 mm, tub2 > por2, pT3(SS), sci, INFc, ly3, v3, pN3a, pPM0, pDM0. (b) Prostate cancer; Adenocarcinoma, Gleason score 4 + 4 = 8 with tertiary pattern 5, pT3a, ly0, v0, pn1, sv0.

Under general anesthesia, the urologists initiated RARP while the patient was on lithotomy position and routinely used six ports. After resection of the prostate and urethrovesical anastomosis, the surgeons inserted additional ports and performed LTG with D2 lymph node dissection, as described in the Japanese classification of GC. Moreover, they extended the umbilical wound and retrieved the resected stomach and prostate. The jejunum that is located 30 cm distal from the ligament of Treitz was transected. After creating a 30-cm Roux-en-Y limb, Y anastomosis was performed via side-to-side jejunojejunostomy. Intracorporeal esophagojejunostomy was carried out using the overlap technique. The surgery lasted for 519 min, and the total volume of blood loss was 250 mL. Oral intake was initiated on postoperative day 2, and the patient was discharged on postoperative day 9. The postoperative course was similar to the standard postoperative course of LTG alone at our hospital. The pathological diagnoses were T3N3aM0 gastric adenocarcinoma and T3aN0M0 prostatic adenocarcinoma (Fig. 3).

Adjuvant chemotherapy with S-1 for GC was started on postoperative day 15. In addition, ADT with bicalutamide for PCa was initiated on postoperative day 27. Six months subsequent to surgery, his body weight decreased only by 3 kg compared to the preoperative weight. He complained of edema in both legs. However, no side effects due to chemotherapy were noted. The patient continually received adjuvant chemotherapy and ADT, and no evidence of cancer recurrence was observed. We planned to continue with adjuvant chemotherapy until 1 year after surgery. Laboratory data and diagnosis obtained via CT scan and ultrasonography will be used for the evaluation of recurrence 1 year after surgery.

## 3 Discussion

Periodic cancer screening and radical resection with regional lymph node dissection have significantly improved the clinical outcomes of patients with GC [3,5]. However, the synchronous presence of another primary malignancy negatively affected the clinical outcomes of GC survivors [5,6]. Kim et al. have hypothesized that synchronous cancers prevented the proper treatment of GC5. To further improve the prognosis, individual cases of GC with other primary malignancies must be discussed.

In terms of technical feasibility and oncological safety, simultaneous laparoscopy combined with resection is the best option for patients with co-existing abdominal lesions [7]. We have previously reported about the advantages of combined laparoscopic and robotic resection for synchronous colorectal cancer and PCa [8]. Based on such experience, combined LTG and RARP were selected.

In Japan, curative gastrectomy is the primary treatment strategy for advanced GC, and adjuvant chemotherapy is required to improve overall and relapse-free survival rates [9]. Multidisciplinary treatment strategies are important in improving quality of life and survival rates in patients with multiple primary cancers [10]. The prompt administration of adjuvant chemotherapy promotes early recovery after surgery. Some studies have reported that combined laparoscopic surgeries can decrease the length of hospital stay and allow for an earlier return in performing daily activities [11,12]. In the present study, adjuvant chemotherapy was initiated on postoperative day 15. Regarding adjuvant chemotherapy, simultaneous endoscopic surgical intervention is advantageous for synchronous GC with another primary cancer.

Currently, in the Japanese guideline, NAC is not recommended for GC with peri-gastric lymph node metastasis alone. A clinical trial of NAC for advanced GC is being conducted, and based on its result, NAC may be recommended. Even in such situation, simultaneous resection is recommended after NAC.

Two-time surgery, such as PARP followed by LTG, may cause GC metastasis due to initial surgical invasion. Various perioperative changes have been proposed to prevent metastases after surgery [13]. Fukaya et al. have reported that immunosuppression due to the first invasive surgery may lead to progression of other untreated cancers [14]. This hypothesis may be a drawback to the two-time surgery for synchronous cancers. For GC with another primary cancer, surgical resection of GC alone may promote the progression of secondary cancer.

The development of metastasis is directly proportional to the magnitude of surgical stress [15,16]. Simultaneous resection of synchronous abdominal lesions is more likely to benefit patients by reducing psychological and physiological stressors correlated to a second surgery [7]. Minimally invasive surgery will play an important role in the improvement of immunosuppression and promotion of early recovery from perioperative invasion.

Currently, except in small facilities, robotic surgery for GC is not covered by the national health insurance program in Japan; therefore, we usually perform laparoscopic surgery. The postoperative course of the patient in the present study was not inferior to that of robotic surgery alone, as reported by Yoo et al. [1].

In a previous study, radical prostatectomy was associated with a lower all-cause mortality with a PSA value >10 ng/mL or with high-risk tumors [17]. However, patients with pT3N0 PCa may experience disease relapse, and radical prostatectomy alone may not be an effective treatment [18]. In Asia, ADT is commonly used as a salvage treatment for recurrence after radical prostatectomy [19]. Significant local control via prostatectomy can improve quality of life, and adjuvant hormone therapy is significantly advantageous in terms of survival [20]. In this case, RARP with adjuvant ADT has been effective in the treatment of locally advanced PCa, and it did not interfere with synchronous GC treatment.

The primary limitation of this study is the fact that it is a single case report. Therefore, large-scale studies must be conducted. At present, treatment for synchronous cancer should be combined with optimal management for individual cancers.

## 4 Conclusion

Synchronous advanced GC and locally advanced PCa were successfully treated. Combined LTG and RARP allowed for minimally invasive radical resection and appropriate adjuvant therapy. Therefore, simultaneous endoscopic surgery is recommended for the treatment of synchronous cancers, including synchronous advanced GC and locally advanced PCa.

## Conflicts of interest

The authors have no conflict of interest to declare.

## Sources of funding

This study did not receive any specific grant from funding agencies in the pubic, commercial, or not-for-profit sectors.

## Ethical approval

This study was approved by the ethics committee in Nagoya Tokushukai General Hospital (Institutional Review Board approval 2019-01-002).

## Consent

Written informed consent was obtained from patient for publication of this case report.

## Author’s contribution

TI, TH, MS and SK performed operation. TI drafted the manuscript. ST participated in the correction of the manuscript. All authors approved the final manuscript.

## Registration of research studies

No research study involved in this case report. Not applicable.

## Guarantor

Toru Imagami.

## Provenance and peer review

Not commissioned, externally peer reviewed.
